# Hypertension Accelerates Alzheimer’s Disease-Related Pathologies in Pigs and 3xTg Mice

**DOI:** 10.3389/fnagi.2018.00073

**Published:** 2018-03-20

**Authors:** Yao-Hsiang Shih, Shih-Ying Wu, Megan Yu, Sheng-Huai Huang, Chu-Wan Lee, Meei-Jyh Jiang, Pao-Yen Lin, Ting-Ting Yang, Yu-Min Kuo

**Affiliations:** ^1^Institute of Basic Medical Sciences, College of Medicine, National Cheng Kung University, Tainan, Taiwan; ^2^Department of Chemistry, University of Virginia, Charlottesville, VA, United States; ^3^Department of Cell Biology and Anatomy, College of Medicine, National Cheng Kung University, Tainan, Taiwan; ^4^Department of Nursing, Ching Kuo Institute of Management and Health, Keelung, Taiwan; ^5^Cardiovascular Research Center, Department of Surgery, College of Medicine, National Cheng Kung University, Tainan, Taiwan; ^6^School of Chinese Medicine for Post Baccalaureate, I-Shou University, Kaohsiung, Taiwan

**Keywords:** hypertension, Alzheimer’s disease, hippocampus, learning and memory, Aβ, tau

## Abstract

Epidemiological studies suggest there is an association between midlife hypertension and increased risk of late-life Alzheimer’s disease (AD). However, whether hypertension accelerates the onset of AD or is a distinct disease that becomes more prevalent with age (comorbidity) remains unclear. This study aimed to test the possible relationship between hypertension and AD pathogenesis. Two animal models were used in this study. For the first model, 7-month-old Lanyu-miniature-pigs were given the abdominal aortic constriction operation to induce hypertension and their AD-related pathologies were assessed at 1, 2, and 3 months after the operation. The results showed that hypertension was detected since 1 month after the operation in the pigs. Levels of Aβ, amyloid precursor protein, RAGE, phosphorylated tau and activated GSK3β in the hippocampi increased at 3 months after the operation. For the second model, 3xTg mice at the ages of 2, 5, and 7 months were subjected to the “two-kidney-one-clip” operation to induce hypertension. One month after the operation, blood pressure was significantly increased in the 3xTg mice in any age. Aβ, amyloid plaque load, and phosphorylated tau levels increased in the operated mice. Furthermore, the operation also induced shrinkage in the dendritic arbor of hippocampal dentate gyrus granule neurons, leakage in the blood-brain barrier, activation in microglia, and impairment in the hippocampus-dependent learning and memory in the 3xTg mice. In conclusion, hypertension accelerates the onset of AD. Blood pressure control during midlife may delay the onset of AD.

## Introduction

Alzheimer’s disease (AD) is an age-related neurodegenerative disease characterized by high densities of amyloid plaques that mainly consisted of amyloid-β (Aβ) and neurofibrillary tangles composed of hyperphosphorylated tau proteins (Probst et al., 1991). Early onset familial forms of AD are associated with autosomal dominant mutations in three genes [amyloid precursor protein (APP), presenilin (PS) 1 and 2] (Selkoe et al., 2012), the elevated production of Aβ peptides, and the presence of neuronal injuries (dystrophic neurites) in the vicinity of amyloid plaques. The amyloid cascade hypothesis, which posits that dementia and AD are induced by Aβ accumulation and deposition, particularly the Aβ42 form (Hardy and Higgins, 1992), has been formulated to describe the etiology of AD. However, the finding that intracellular phosphorylated tau (p-tau) and neurofibrillary tangles, but not the locations of Aβ/amyloid, correspond to the pathology and cognitive symptoms of the early phase of AD has contested this hypothesis (Braak et al., 1998; Desikan et al., 2011). The etiology of the late-onset sporadic form of AD remains unclear.

Cardiovascular diseases and AD share many common risk factors, including diabetes, high serum cholesterol, atherosclerosis, and hypertension (de la Torre, 2004; Birkenhager and Staessen, 2006; Purnell et al., 2009). Among them, midlife hypertension (HTN) is recognized as a strong AD risk factor (Birkenhager and Staessen, 2006; Baumgart et al., 2015). HTN changes the integrity of cerebral vasculatures (Kaiser et al., 2014), promotes atherosclerosis in cerebral arteries, and induces lipohyalinosis (Droste et al., 2003; Rosenblum, 2008), and is associated with cognitive decline, dementia, and the onset and progression of AD (Skoog et al., 1996; Kivipelto et al., 2001; Baumgart et al., 2015). The use of antihypertensive drugs reduces the risk of dementia (Haag et al., 2009) and slows cognitive decline in AD patients (Furiya et al., 2013), and midlife HTN has been associated with increased densities of amyloid plaques, cerebral amyloid angiopathy, and neurofibrillary tangles in postmortem brains (Shah et al., 2012). Collectively, these findings suggest an association between HTN and AD. Whether HTN accelerates the development of late-onset AD or is a comorbid illness that becomes more frequent with age remains unclear. It has been shown that HTN-associated memory impairment is primarily caused by comorbid conditions, such as diabetes and aging (Albert, 1993; Elias et al., 1997). After adjusting for the comorbidities, the association between HTN and memory impairment became statistically insignificant.

This study aimed to test the possible relationship between HTN and late-onset AD. We adopted two established HTN animal models: (1) non-genetic modified pigs and (2) mice that overexpress three AD-related mutant genes (*Psen1*^tm1Mpm^/*APP*_Swe_/*TauP301L*) (also called 3xTg mice) (Oddo et al., 2003) to examine the role of HTN in AD pathogenesis. We chose the pig as one of the animal models, because pigs and humans share the same Aβ amino acid sequence (Johnstone et al., 1991), and AD-related pathologies are evident in non-genetic modified pigs (Smith et al., 1999). Furthermore, the cardiovascular systems in humans and pigs are similar in structure and function (Crick et al., 1998). Here, we adopted the abdominal aortic constriction (AAC) method (Lin et al., 2013) to induce HTN in pigs and the two-kidney one-clip (2K1C) method (Lorenz et al., 2011; Shih et al., 2016) to induce HTN in the frequently used AD transgenic mouse model, 3xTg mice. Blood pressure and the effects of HTN on AD-related pathologies and blood-brain barrier (BBB) integrity were examined in both pigs and 3xTg mice. Learning and memory performance were evaluated in the 3xTg mice.

## Materials and Methods

### Animals

All experiments were done in accordance with the National Institutes of Health Guideline for Animal Research (Guide for the Care and Use of Laboratory Animals) and Taiwan Animal Protection Law, and were approved by the National Cheng Kung University Institutional Animal Care and Use Committee.

Non-genetic modified Lanyu miniature pigs (*Sus barbatus sumatranus*), an established pig strain for cardiovascular research (Lin et al., 1999), were obtained from the Taitung Animal Propagation Station of the Taiwan Livestock Research Institute (Taitung, Taiwan). All pigs were quarantined before experiments. This strain of pig was registered as a formal breed according to the regulations formulated by the Council of Agriculture of Taiwan (breed code: Lanyu 300/Lanyu GPI-CRC-PGD Line). The average lifespan of Lanyu miniature pigs is 10–15 years. They reach sexual maturity at the age of 4–5 months. Further information on Taiwanese Lanyu pigs can be found online website^1^. Pigs were housed in a semi-open environment in the Livestock Research Institute, located at the Council of Agriculture in Tainan city, Taiwan. Each pig was housed individually in a cage supplied with 1.8 kg of hog fattening meal (prepared in house) each day and free access to water.

Male 3xTg mice [B6; 129-Psen1tm1Mpm Tg (APPSwe, TauP301L) 1Lfa/Mmjax)] expressing three mutant genes (*Psen1*^tm1Mpm^/*APP*_Swe_/*TauP301L*) were obtained from the Jackson Laboratory (Bar Harbor, ME, United States) and housed (4–5 per cage) at a stable temperature (23 ± 1°C), a 12 h light/dark cycle, and unrestricted access to food and water. All mice were genotyped using a protocol provided by the Jackson Laboratory^2^.

### Pig HTN Induced by Abdominal Aortic Constriction (AAC)

The AAC method used to induce HTN in pigs has been previously described (Lin et al., 2013). Male 7-month-old pigs (average body weight = 50 ± 10 kg) were used in this study. Pigs were anesthetized by intramuscular injection of a mixture of tranquilizer (Zoleti 50, 10 ml, Vibrac, Amherst, MA, United States), analgesics (Rompun, 5 ml, Bayer, Toronto, ON, Canada) and anticholinergic compound (Atropine, 1 ml, Shinlon, Tainan, Taiwan). Endotracheal intubation was performed and immediately connected to a ventilator NAD-2B (Narkomed, DRE, Louisville, KY, United States) with 20% O_2_ and 2% (tidal volume) isoflurane. Aortic constriction was achieved by circumferentially wrapping an ePTFE Teflon strip around a segment of the infrarenal abdominal aorta, approximately 2 cm away from the bifurcation of common iliac arteries, to make the outside external diameter of abdominal aorta 8 mm (Lin et al., 2013). In the sham procedure, a Teflon strip was wrapped around the abdominal aorta without aortic constriction. Heart rate, blood pressure and blood oxygen concentration were recorded during surgery and at post-operative months (POMs) 1, 2, and 3 using the physiological monitor system PAMO II (MEK-ICS, Seongnam-si, Seoul, Korea). This AAC procedure reduces the pulsatility index [(maximal flow rate - minimal flow rate)/mean flow rate] to about one-third of the original level, meeting the criteria of moderate constriction (Lin et al., 2013).

### Mouse HTN Induced by the Two-Kidney One-Clip (2K1C) Method

The 2K1C method, used to induce HTN in mice, was previously described (Lorenz et al., 2011; Shih et al., 2016). This method constricts one renal artery and activates the renin-angiotensin-aldosterone system to induce hypertension (Wiesel et al., 1997). To achieve renal artery constriction, the left renal artery was isolated and a 1-mm polyethylene tubing (SP8, O.D. 0.5 mm, I.D. 0.12 mm, Natsume Seisakusho, Tokyo) was cut open and placed around the left renal artery (Shih et al., 2016). The tubing was tied with a 6–0 silk suture in order to maintain the position and diameter of the clip. Sham control mice underwent the same surgical procedure without the clip placement. The blood pressure of these mice was measured using tail-cuff plethysmography (BP-2000 system, VisiTech Systems, Apex, NC, United States).

Because the typical onset age of memory impairment of the 3xTg mice is about 5-month-old (Lee and Han, 2013), the 2K1C operation was performed before (2-month-old), during (5-month-old) and after (7-month-old) the onset age. Mice were examined for their learning and memory and AD-related pathologies at either POM 1 or POM 3.

### Object Recognition Test

The object recognition test was used to determine the hippocampus-dependent non-spatial learning and memory (Leger et al., 2013). Each mouse was allowed to freely explore a polycarbonate box (48 cm × 26 cm × 21 cm) 10 min per day for two consecutive days (habituation phase). On the third day, the mouse was placed in the same box for 5 min that contained two identical objects (transparent glass bottles, diameter = 5 cm, height = 9.5 cm) separately positioned 10 cm away from a wall (acquisition phase). Two hours later, the mouse was placed for 5 min in the same box; one of the old objects was replaced with a new object (white plastic box: 3 cm × 7.5 cm × 8 cm) (short-term task phase). The time spent to explore each object was recorded. Short-term memory was determined by calculating the ratio of “new-object exploration time divided by total exploration time.” Twenty-two hours after the short-term task phase (24 h after the acquisition phase), the mouse was again placed for 5 min in the same box; the initially new object was replaced with a different new object (stainless box, 4 cm × 6.5 cm × 7.5 cm) (long-term task phase). We defined exploration behavior as the phenomenon in which the mice touch the object with the nose or sniff the object within a distance of 1 cm. Long-term memory was determined by calculating the ratio of “newest object exploration time divided by total exploration time.”

### Radial Arm Water Maze

The radial arm water maze test was performed in a circular pool (diameter = 120 cm; wall height = 60 cm), and this procedure was described elsewhere (Shukitt-Hale et al., 2004). The pool was filled to a depth of 25 cm with clear, 24 ± 1°C tap water. In the center of the pool, a 6-arm, white, acrylic maze (each arm length = 44 cm; wall height = 30 cm) was arranged to make six swim paths with each arm radiating from an open center area. The square (10 × 10 cm) escape platform made of transparent Plexiglas was placed at the end of one arm, submerged 2 cm below the surface of the water. The mice were given three training trials beginning at 4 _PM_ per day for 4 days. During every spatial navigation trial, the location of the hidden platform was kept constant. The mice were randomly placed on one of the five arms that did not contain the platform, and they were placed on a different arm in every trial (Shukitt-Hale et al., 2004). Each mouse was allowed a maximum of 120 s to escape onto the platform; if the mouse failed to escape within this time frame, it was guided to the platform. Once the mouse reached the platform, it remained there for 30 s. The platform was removed on day 5 for the probe test. The whole process was recorded using a CCD camera; the escape latency (i.e., the time in seconds to reach the platform), errors (numbers in entering an arm that does not contain the platform), path length, and swim speed (cm/s) were analyzed using a video tracking system (EthoVision; Noldus Information Technology, Wageningen, Netherlands).

### Tissue Preparation

The pigs were anesthetized using the same method as for the AAC operation and then sacrificed with an intravenous injection of KCl (2 mM/Kg) as previously described (Lin et al., 2013). The mice were anesthetized using urethane (1.5 g/kg, i.p.) and perfused from the left ventricle with ice-cold PBS, and their brains were quickly removed. The olfactory bulb, cortex, thalamus, hippocampus, brain stem and cerebellum of the pigs and mice were isolated from their left hemispheres. For the pig brains, we used the recessus preopticus-posterior commissural line as the landmark to define the origin of the stereotaxic atlas (Felix et al., 1999). The anterior limit of the posterior commissural was used as the zero coordinate of horizontal plane landmark, and the sagittal suture was used as the zero coordinate of sagittal plane landmark. The pig hippocampi were further divided into dorsal (stereotaxic coordinates in mm: anterior/posterior, -3 ∼-4.5; lateral, +2 ∼ +9; horizontal, +11 ∼ +16) and ventral (anterior/posterior, 0 ∼ +4; lateral, +10 ∼ +20; horizontal, +5 ∼-10) parts (Felix et al., 1999). The mouse hippocampi were also divided into dorsal (stereotaxic coordinates in mm from bregma: anterior/posterior, -1.1 ∼-2.0) and ventral (anterior/posterior, -3.0 ∼-3.8) parts. The region located between the stereotaxic coordinates -3.9 to -4.3 mm was defined as posterior CA region. The posterior CA region lacks the granule cells of dentate gyrus and pyramidal cells of CA layer. The brain tissues were stored at -70°C until use. The right hemispheres were fixed in 4% buffered paraformaldehyde at 4°C ready for frozen section (see Immunohistochemistry section below).

### Aβ Quantification

The concentrations of Aβ40 and Aβ42 were determined by using commercial ELISA kits (Human Aβ40 and Aβ42 ELISA Kits, MyBioSource, San Diego, CA, United States). According to the manufacturer’s manual, there is no significant cross-reactivity or interference between human Aβ and other analogs. Because the amino acid sequences of human Aβ and pig Aβ are identical, these kits are expected to detect Aβ peptides in pig brain homogenates. In the brain homogenates of 3xTg mice, these kits are expected to detect the overexpressed human Aβ peptides, but not the mouse Aβ peptides which have 3 amino acid differences at residues 5 (R→G), 10 (Y→F) and 13 (H→R) from human counterparts.

The brain tissues were homogenized (1:8, w:v) in 5 M guanidine HCl/50 mM Tris-HCl. The homogenates were gently mixed for 4 h before dilution (mouse 100x, pig 20x) in a buffer containing 0.2 g/L KCl, 0.2 g/L KH_2_PO_4_, 8.0 g/L NaCl, 1.15 g/L Na_2_HPO_4_, 5% BSA and 0.03% Tween-20. The diluents were centrifuged at 15,000 × *g* for 1 h at 4°C. The supernatants were applied to the 96-well plate, and results were read in a spectrophotometer reader (μQuant, Bio-Tek Instruments, Winooski, VT, United States) at an absorbance wavelength of 450 nm. Standard curves were obtained from values generated from known concentrations of Aβ provided by the kits.

### Thioflavin-S Staining

Mounted slices were incubated with 0.2% (w/v) thioflavin S in PBS for 20 min, and then washed three times with PBS. The sections were washed in 50% ethanol and in distilled water, dried, and dipped into Histo-Clear before being mounted with mounting gel.

### Western Blotting

We performed Western blots to determine the relative levels of proteins of interest. Frozen brain tissues were homogenized by sonication in a lysis buffer containing 50 mM Tris-HCl, pH 7.4, 10 mM EDTA, 1% SDS, 0.5% Triton X-100, and protease inhibitors (Roche Diagnostics, Mannheim, Germany). The homogenates were centrifuged at 15,000 × *g* for 30 min at 4°C, and the protein concentrations of the supernatants were determined and adjusted to the same concentration. Supernatants (30 μg of total protein) were mixed with the sample buffer, which contained 0.5 M of dithiothreitol. This mixture was then heated to 75°C for 15 min, loaded into each well of 4–12% polyacrylamide gel (Nu-PAGE gel; Invitrogen, Camarillo, CA, United States), and resolved at 120 V for 1 h. The separated proteins were transferred to a nitrocellulose membrane (Bio-Rad Laboratories, Hercules, CA, United States), blocked with 5% non-fat milk, and probed with the following primary antibodies: mouse monoclonal anti-Alzheimer precursor protein A4 antibody (1:5000, Merck Millipore, Darmstadt, Germany), rabbit polyclonal anti-tau pS262 phospho-specific antibody (1:1000, Invitrogen), and rabbit polyclonal anti-tau pS412 phosphospecific antibody (1:1000, Anaspec, Fremont, CA, United States). The amino acid sequences of human and pig tau at the three selected tau phosphorylation sites (T212, S262 and S412) and nearby antibody recognizing epitopes are identical (please see their sequences comparison below). Hence, phosphorylation at these three positions in pig tau should also be detected by the anti-human tau antibodies. A monoclonal anti-β-actin antibody (1:10000, Merck Millipore) served as the control for protein loading. The bound antibodies were detected by using an enhanced chemiluminescence detection kit (PerkinElmer, Boston, MA, United States). The band densities were measured using an imaging system (BioChemi; UVP, Upland, CA, United States), and analyzed using ImageJ (version 1.43)^3^.

### Immunohistochemistry

The detailed procedure for immunohistochemistry has been described elsewhere (Shih et al., 2016). In short, coronal sections (30 μm thickness) of the right hemisphere were blocked with goat serum (3% in PBS/0.5% Triton X-100) for 1 h, and probed with the following primary antibodies: pS412-tau (1:1000, AS-55418P, Anaspec), rabbit anti-ionized calcium binding adaptor molecule 1 (Iba1) (1:1000, 091-19741, Wako, Japan) for microglia and biotinylated goat anti-mouse IgG (1:1500, BA-9200, Vector Laboratories, Burlingame, CA, United States) for BBB leakage. The floating sections were incubated with primary antibodies for 16 h at 4°C, then incubated with appropriate secondary antibodies (Vector Laboratories) and avidin-biotin peroxidase (Vector) using 3, 3′-diaminobenzidine as the substrate (Shih et al., 2016). A parallel section stained without primary antibody served as the negative control.

### Golgi-Cox Stain

The mice were perfused with 0.1 M PBS at POM 1 and their brains were quickly removed and incubated in Golgi-Cox solution (5% K_2_CrO_4_, 5% K_2_Cr_2_O_7_, and 5% HgCl_2_ in ddH_2_O) for 2 days in dark as previously described (Tsai et al., 2016). The brains were then transferred to a fresh Golgi-Cox solution for additional 14 days before dehydration in 30% sucrose solution for 2 days. Coronal sections (200 μm thickness) were cut using vibratome. Three brain sections per animal containing the septal hippocampus (1.5–2.1 mm posterior to Bregma) were transferred to 50% NH_4_OH for 30 min, rinsed in ddH_2_O, and transferred to fix solution (8x diluent, Kodak, Rochester, NY, United States) for 30 min. Finally, the sections were serially dehydrated in ethanol, incubated in Xylene for 10 min, and mounted with mounting gel.

### Neuronal Morphometry

The procedure for assessing the effect of 2K1C on dendritic morphology and spine density of the granule neurons in the hippocampal dentate gyrus was previously described (Chen et al., 2011; Wang et al., 2011). The protrusion emerging from the neurite with a length from 0.5 to 5 μm serve as the single dendritic spine. Protrusions longer than 10 μm were recognized as dendritic branches. Morphometric measurements were performed using ImageJ (version 1.43u, NIH, United States). The number of spines on different branch orders of dendrites (from order 1 to order 3) were manually counted and traced. Only the dominant neurite, which is at least twofold longer than the rest of individual neurites, was used to determine the different order neurite length. To analyze the neural morphometry, photomicrographs were taken using an AxiocamMRc digital camera connected to a computer equipped with Axiovision 4.8 software (Carl Zeiss, Oberkochen, Germany). For 3D measurement, the image was acquired as a Z-series of 20–30 sections spaced 1 μm apart. For publication, the Z-series were projected into single images using ImageJ with minimal adjustment of brightness or contrast applied to the whole image.

For the neural morphometric study, five mice were used in each Sham and 2K1C group. Three to five neurons from each animal were labeled and measured in regions of interest, and the average value of each parameter was used as one individual animal.

### Quantifying pS412-tau, IgG and NeuN Signal

The entire hippocampus of a mouse was cut into an average of 130 30-μm coronal sections. The intensities of pS412-tau-positive and IgG-positive signals were measured in every 12th section of the entire hippocampus. Photomicrographs were taken using a digital camera (Axiocam MRc; Carl Zeiss, Oberkochen, Germany) connected to a computer equipped with imaging software (Axiovision 4.8; Carl Zeiss). Signal relative intensities were obtained by dividing the optical densities of the desired brain regions to fornix part of each mouse using the ImageJ software (version 1.49 m, NIH, United States). The optical densities in the Sham group were set as 1 and the optical densities in the AAC and 2K1C groups were adjusted accordingly.

### Quantifying Microglial Area and Density

Photomicrographs were taken using a digital camera, and the Iba1^+^ areas were obtained using image analysis software (Image-Pro Plus 6.0) by measuring the areas with Iba1^+^ intensities higher than a given background threshold. The background intensity threshold was fixed, and used for all sections.

The number of Iba1^+^ cells was counted using a modified stereological procedure (Yang et al., 2013). Briefly, the selected region was first outlined under a 10x objective lenses, and the number of positively stained cells were counted using the optical dissection (1 μm per section) method through a computer-controlled x-y-z motorized stage and a 100x oil immersion objective (Carl Zeiss). Cell counting began 2 μm below the top of the section. The number of labeled cells per section was divided by the area to obtain the cell density.

### Statistical Analysis

Results are presented as mean ± standard deviation. Significance was set at *p* < 0.05. Two-way ANOVA was used to analyze the main effects of HTN and POM and any possible interaction between them. Bonferroni *post hoc* tests were performed for any significant main effect or interaction. Univariate correlations between blood pressure and levels of p-tau, pGSK3β, pAKT and Aβ were assessed by Pearson’s correlation.

## Results

### AAC Induces HTN in the Pigs

**FIGURE 1:**
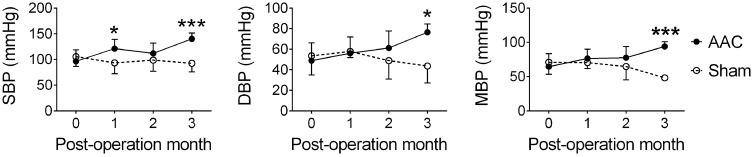
Abdominal aortic constriction (AAC) increased blood pressure in the pigs. 7-month-old pigs were given the AAC or the Sham operation, and their blood pressure was measured before and 1, 2, and 3 months post-operation. SBP, systolic blood pressure; DBP, diastolic blood pressure; MBP, mean blood pressure. ^∗^*p* < 0.05, ^∗∗∗^*p* < 0.001 versus respective Sham group, Bonferroni’s *post hoc* test. *N* = POM 0: Sham: 21, AAC: 27; POM 1: Sham: 8, AAC: 10; POM 2: Sham: 8, AAC: 10; POM 3: Sham: 5, AAC: 7.

The infrarenal AAC method was used to induce HTN in the 7-month-old pigs (Lin et al., 2013), and their suprarenal abdominal aorta blood pressure was measured at POMs 1, 2, and 3. AAC significantly altered the systolic blood pressures (*F* = 16.6, *p* < 0.001), diastolic blood pressures (*F* = 6.0, *p* = 0.016), and mean blood pressures (*F* = 13.6, *p* < 0.001) (**Figure 1**). *Post hoc* analyses showed that the systolic, diastolic, and mean blood pressures of the AAC pigs were significantly higher than those of Sham pigs on POM 3.

### AAC Increases Levels of Aβ and Microglial Activation in the Hippocampi of Pigs

**FIGURE 2:**
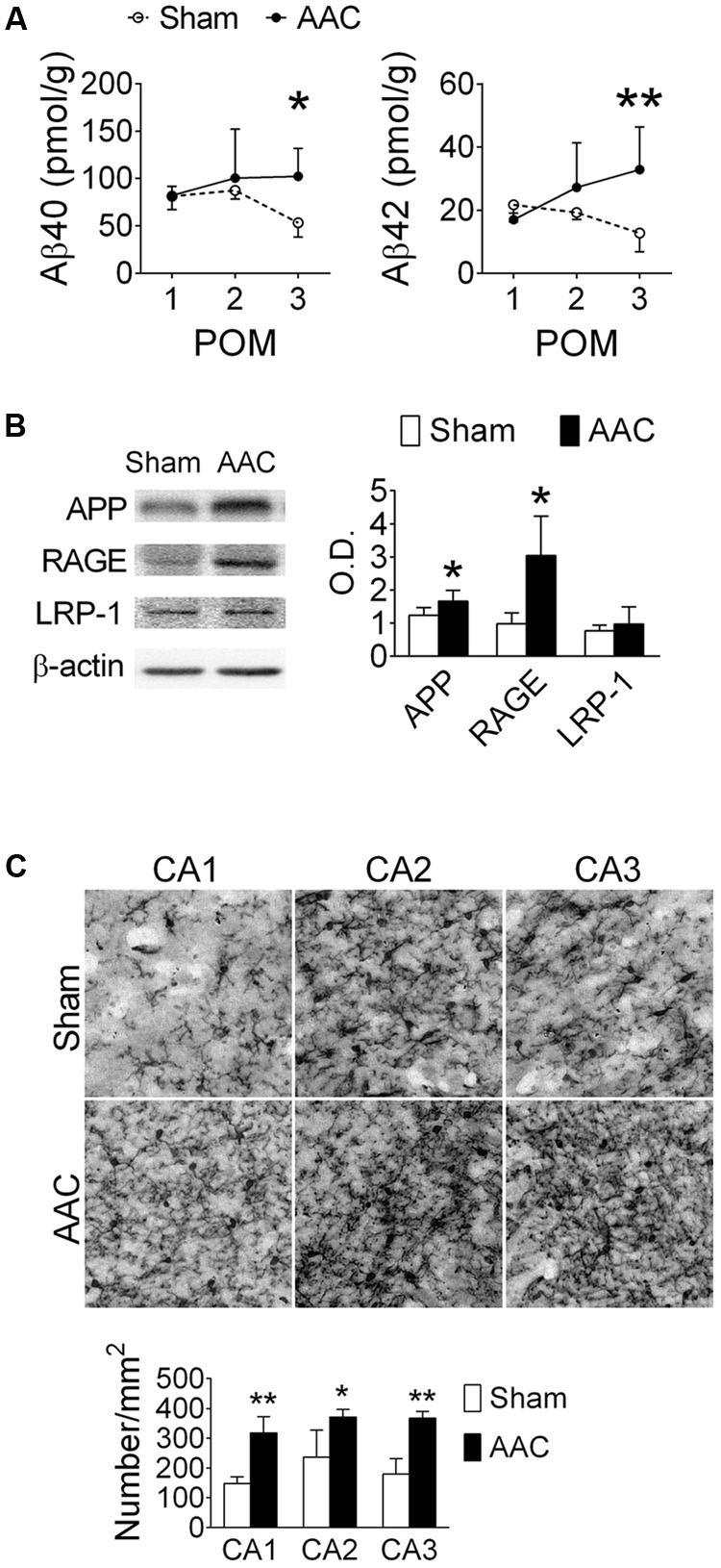
Abdominal aortic constriction increased levels of Aβ and microglial activation in the hippocampi of pigs. 7-month-old pigs were given the AAC or the Sham operation, and their hippocampi were removed at POMs 1, 2, and 3 for analyses. **(A)** Concentrations of soluble Aβ40 and Aβ42. *N* = POM 1: Sham: 4, AAC: 5; POM 2: Sham: 5, AAC: 5; POM 3: Sham: 5, AAC: 5. **(B)** Relative levels of APP, RAGE and LRP-1 in the POM 3 pigs. Representative immunoblotting micrographs are shown on the left panels; the quantitative results are shown on the right panel. *N* = Sham: 6, AAC: 7. Full-length blots are presented in **Supplementary Figure S2**. **(C)** Status of microglial activation in the POM 3 pigs. Representative Iba1 immunostaining micrographs are shown on the top panels. The densities of Iba-1-positive microglial cells are shown on the lower panel. *N* = Sham: 3, AAC: 3. ^∗^*p* < 0.05, ^∗∗^*p* < 0.01 versus respective Sham group, Bonferroni’s *post hoc* test.

The effect of AAC on hippocampal Aβ concentrations in the pigs was determined at POMs 1, 2, and 3. Hippocampal Aβ40 and Aβ42 concentrations gradually increased after AAC operation and became significantly higher than those of the Sham group on POM 3 (**Figure 2A**). We did not observe any Aβ-positive plaque-like staining in the brains of Sham or AAC POM 3 pigs. On POM 3, the levels of APP and receptor of advanced glycation end product (RAGE), which regulates the influx of Aβ (Deane et al., 2003), increased in the hippocampi of the AAC pigs while the levels of low-density lipoprotein receptor-related protein 1 (LRP-1), which mediates the efflux of Aβ (Shibata et al., 2000), did not change (**Figure 2B**).

The effect of AAC on microglial activation was determined by the immunostaining of Iba-1 on POM 3. The intensities of the Iba-1-positive signal increased in the hippocampi of AAC pigs (**Figure 2C**). Iba-1-positive microglial cells became denser in three hippocampal CA regions of the AAC pigs (*F* = 47.0, *p* < 0.001).

### AAC Increases Levels of Phosphorylated Tau Protein in the Hippocampi of Pigs

**FIGURE 3:**
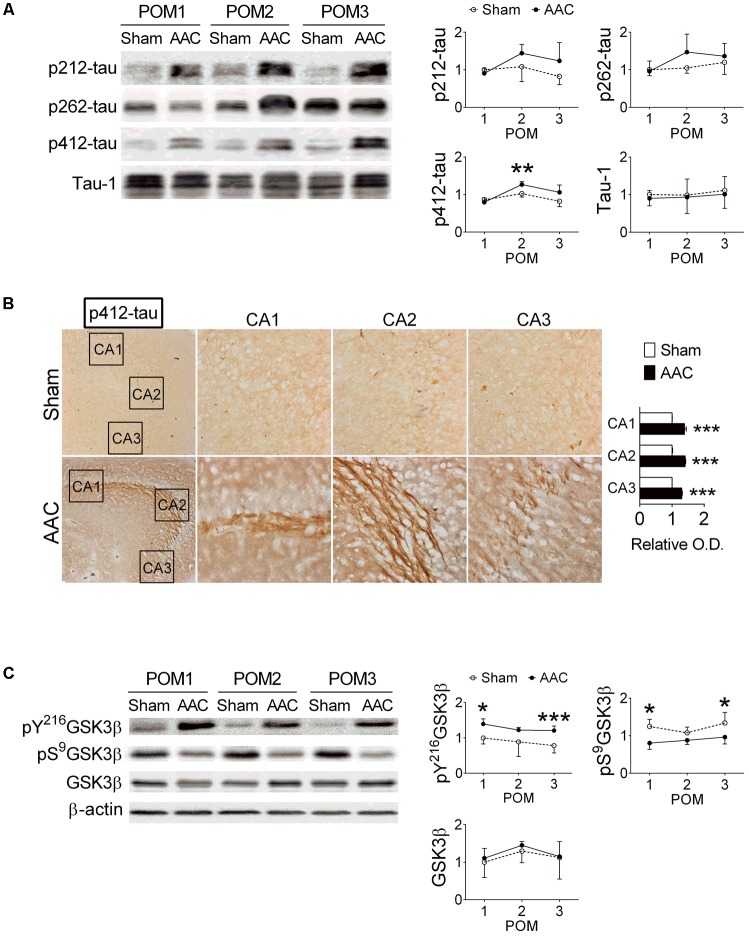
Abdominal aortic constriction increased levels of phosphorylated tau protein in the hippocampi of pigs. 7-month-old pigs were given the AAC or the Sham operation, and their hippocampi at POMs 1, 2, and 3 were removed for analyses. **(A)** Relative levels of p212-, p262-, p412-, and total tau (Tau-1). Representative immunoblotting micrographs are shown on the left panels; the quantitative results are shown on the right panels. *N* = POM 1: Sham: 3, AAC: 4; POM 2: Sham: 3, AAC: 5; POM 3: Sham: 6, AAC: 7. **(B)** Expression of p412-tau in the hippocampi of POM 3 pigs. Representative immunostaining micrographs are shown on the left panels; the quantitative results of relative optical density (O.D.) are shown on the right panel. *N* = Sham: 3, AAC: 3. **(C)** Relative levels of pY^216^GSK3β, pS^9^GSK3β, GSK3β, and β-actin. Representative immunoblotting micrographs are shown on the left panels; the quantitative results are shown on the right panels. *N* = POM 1: Sham: 3, AAC: 4; POM 2: Sham: 3, AAC: 5; POM 3: Sham: 6, AAC: 7. ^∗^*p* < 0.05, ^∗∗^*p* < 0.01, ^∗∗∗^*p* < 0.001 versus respective Sham group, Bonferroni’s *post hoc* test. Full-length blots are presented in **Supplementary Figure S3**.

The relative levels of p-tau in the hippocampi were determined at POMs 1, 2, and 3 (**Figure 3A**). We focused on Thr212, Ser262 and Ser412 of tau because their phosphorylation was previously correlated with severities of neuronal pathology in AD (Augustinack et al., 2002; Hanger et al., 2007). Among them, the levels of pS412-tau were increased after AAC surgery (*F* = 6.2, *p* = 0.021); whereas the levels of pT212-tau (*p* = 0.091), pS262-tau (*p* = 0.186) and total tau (Tau-1) (*p* > 0.5) did not significantly change (**Figure 3A**).

The expressions of pT212-, pS262- and pS412-tau in the hippocampal sub-regions of AAC pigs were also examined at POM 3. Two-way ANOVA revealed that AAC significantly increased the intensities of pS412-tau staining signals (*F* = 1069, *p* < 0.001) (**Figure 3B**), and to a less extend the intensities of pT212-tau (*F* = 5.6, *p* = 0.035) and pS262-tau (*F* = 14.4, *p* = 0.002) (**Supplementary Figure S1**). *Post hoc* analyses revealed that the pS412-tau staining signals in all three hippocampal sub-regions of AAC pigs were more intense than those of Sham pigs (**Figure 3B**); whereas, the intensities of the pT212-tau staining signals were only increased in the CA1 region and pS262-tau only in the CA3 region (**Supplementary Figure S1**).

Because the GSK3 signaling pathway has been suggested as one of the major regulators of tau phosphorylation (Baki et al., 2004), the effect of AAC on the GSK3 pathway was studied. AAC increased the levels of the active form of GSK3β, pY^216^GSK3β (*F* = 25.3, *p* < 0.001), but decreased the levels of the inactive form of GSK3β, pS^9^GSK3β (*F* = 30.3, *p* < 0.001), in the hippocampi (**Figure 3C**). The total levels of GSK3β were unaltered (*p* > 0.5).

### The 2K1C Method Induces HTN in the 3xTg Mice

**FIGURE 4:**
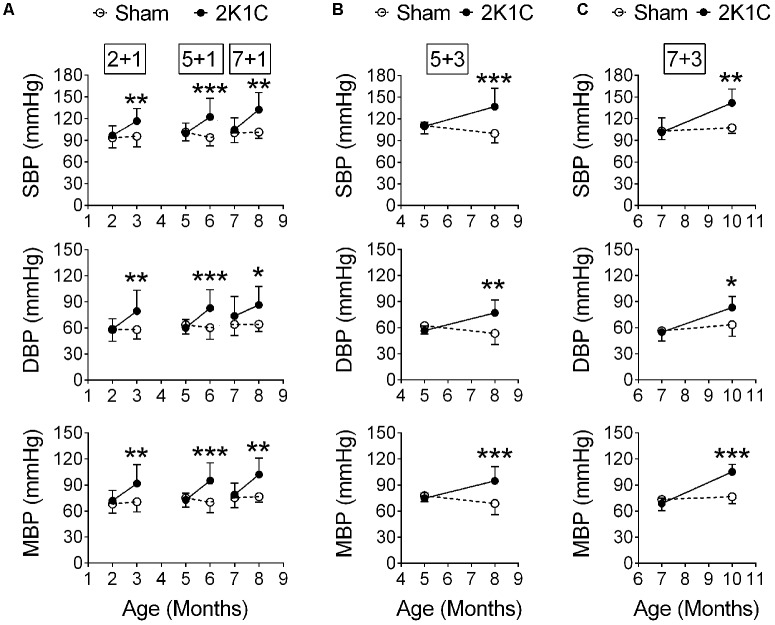
2K1C increased blood pressures in the 3xTg mice. 2-, 5-, and 7-month-old mice were given the 2K1C or the Sham operation, and their blood pressure was measured. **(A)** Blood pressure right before and 1 month post-operation (termed 2 + 1, 5 + 1, and 7 + 1 groups). **(B,C)** Blood pressure right before and 3 month post-operation (termed 5 + 3 and 7 + 3 groups). **(B)** 5 + 3 groups. *N* = Sham: 7, 2K1C: 6. **(C)** 7 + 3 groups. *N* = Sham: 5, 2K1C: 5. SBP, systolic blood pressure; DBP, diastolic blood pressure; MBP, mean blood pressure. ^∗^*p* < 0.05, ^∗∗^*p* < 0.01, ^∗∗∗^*p* < 0.001 versus respective Sham group, Bonferroni’s *post hoc* test.

The 2K1C operation was performed before (2-month-old), during (5-month-old) and after (7-month-old) the typical onset age of memory impairment of the 3xTg mice (Lee and Han, 2013). Their blood pressures were measured at POM 1 (hereafter termed 2 + 1, 5 + 1, and 7 + 1 groups, respectively). Two-way ANOVA revealed that 2K1C significantly increased the systolic, diastolic, and mean blood pressures on POM 1 (**Figure 4A**). In a separate batch, 5- and 7-month-old 3xTg mice were given the 2K1C operation, and their blood pressures were measured at POM 3 (hereafter termed 5 + 3 and 7 + 3 groups, respectively). The systolic, diastolic, and mean blood pressures of the 5 + 3 (**Figure 4B**) and 7 + 3 (**Figure 4C**) 2K1C groups were significantly higher than those of 5 + 3 and 7 + 3 Sham groups, respectively.

### 2K1C Increases Levels of Aβ, Amyloid Depositions and Microglial Activation in the Hippocampi of 3xTg Mice

**FIGURE 5:**
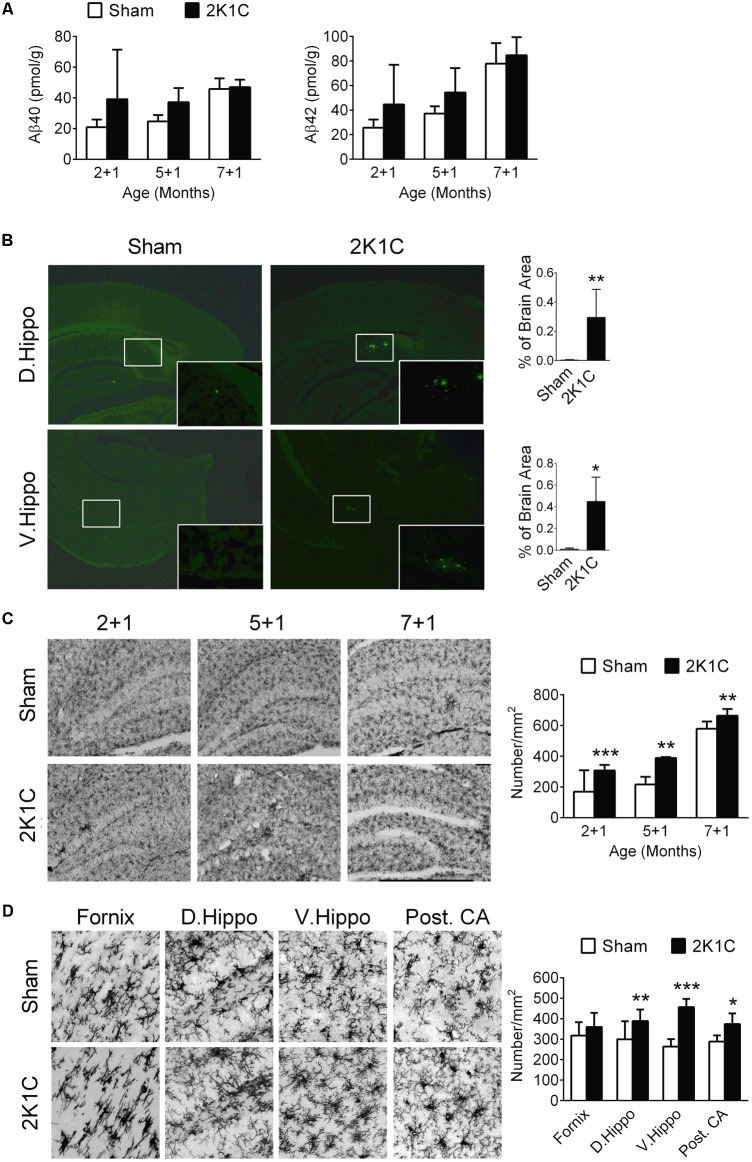
2K1C increased levels Aβ, amyloid depositions and microglial activation in the hippocampal of 3xTg mice. 2-, 5-, and 7-month-old mice were given the 2K1C or the Sham operation, and their hippocampi were removed for analyses. **(A)** Hippocampal concentrations of soluble Aβ40 and Aβ42 at 1 month post-operation (termed 2 + 1, 5 + 1, and 7 + 1 groups). *N* = 2 + 1: Sham: 8, 2K1C: 3; 5 + 1: Sham: 3, 2K1C: 3; 7 + 1: Sham: 6, 2K1C: 4. **(B)** Thioflavin-S-positive amyloid plaques in the hippocampi of 7 + 3 groups. Thioflavin-S staining micrographs are shown on the left panels; the quantitative results are shown on the right panels. *N* = Sham: 5, 2K1C: 5. ^∗^*p* < 0.05, ^∗∗^*p* < 0.01 versus respective Sham group, two-tailed *t*-test. **(C)** Representative Iba1 immunostaining micrographs are shown on the left panels. The densities of Iba-1-positive microglial cells are shown on the right panel. *N* = 6 for each group. ^∗∗^*p* < 0.01, ^∗∗∗^*p* < 0.001 versus respective Sham group, Bonferroni’s *post hoc* test. **(D)** Expression of Iba-1-positive microglial cells in the hippocampal subregions of the 7 + 3 mice. *N* = 6 for each group. ^∗^*p* < 0.05, ^∗∗^*p* < 0.01, ^∗∗∗^*p* < 0.001 versus respective Sham group, two-tailed *t*-test. D. Hippo, dorsal part of hippocampus; V. Hippo, ventral part of hippocampus; Post. CA, posterior CA part of hippocampus.

In the three POM 1 (2 + 1, 5 + 1, and 7 + 1) groups, both age and 2K1C significantly influenced the concentrations of Aβ40 and Aβ42 (**Figure 5A**). *Post hoc* analyses revealed that the effects of 2K1C on the concentrations of Aβ40 and Aβ42 were more pronounced at younger ages.

To evaluate the effect of 2K1C on amyloid plaque deposition, brain sections of the 7 + 3 group were stained by thioflavin-S (**Figure 5B**). This age was chosen because in the 3xTg mice, deposition of amyloid plaque typically starts at about 9-month-old (Oddo et al., 2003). The areas of thioflavin-S, positively stained amyloid plaques were significantly increased in the dorsal and ventral portions of the hippocampi and nearby subiculum regions of the 2K1C mice (**Figure 5B**).

The densities of Iba-1-positive microglial cells in the hippocampi were significantly affected by age (*F* = 102.4, *p* < 0.001) and 2K1C treatment (*F* = 42.7, *p* < 0.001) (**Figure 5C**). *Post-hoc* analyses revealed that the densities of Iba-1-positive cells of the 2K1C mice were higher than those of Sham mice in all three age groups. Morphologically, the Iba-1-positive cells in the 7 + 3 2K1C mice showed thickened/retracted branches and enlarged cell bodies while microglia in the hippocampi of the Sham mice typically existed in ramified morphology (**Figure 5D**). The densities of Iba-1-positive microglial cells in the dorsal, ventral, and posterior CA parts of the hippocampi of the 2K1C mice were higher than those of the Sham mice (**Figure 5D**). These changes were not evident in the fornix.

### 2K1C Increases Levels of Phosphorylated Tau in the Hippocampi of 3xTg Mice

**FIGURE 6:**
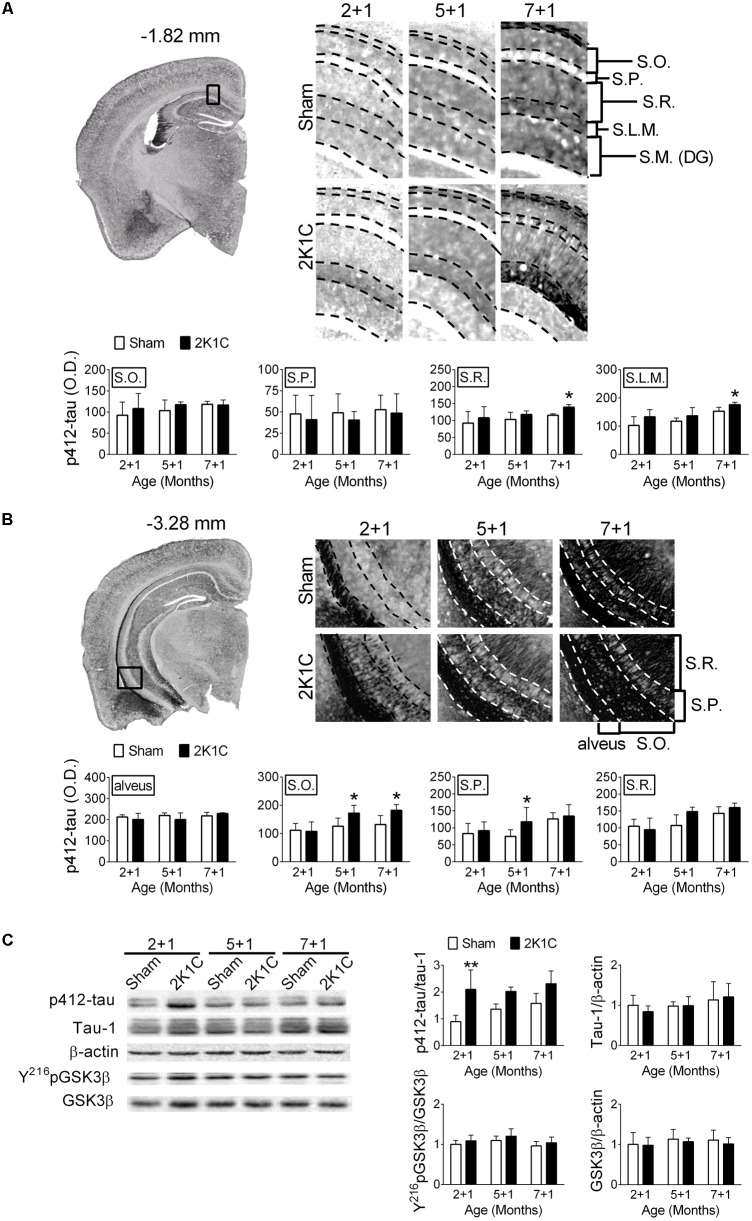
2K1C increased hippocampal levels of phosphorylated tau in the 3xTg mice. 2-, 5-, and 7-month-old mice were given the 2K1C or the Sham operation, and their brains were removed at 1 month post-operation (termed 2 + 1, 5 + 1, and 7 + 1 groups). Expressions of p412-tau in the dorsal **(A)** and ventral **(B)** parts of hippocampi. Representative immunostaining micrographs are shown on the top left panel. The boxed regions are enlarged and shown on the top right panels. The quantitative results are shown on the lower panels. *N* = 2 + 1: Sham: 4, 2K1C: 5; 5 + 1: Sham: 5, 2K1C: 4; 7 + 1: Sham: 4, 2K1C: 5. S.O., stratum oriens; S.P., stratum pyramidale; S.R., stratum radiatum; S.L.M., stratum lacunosum-moleculare; S.M. (DG), stratum moleculare of the dentate gyrus. **(C)** Relative levels of p412-tau, total (Tau-1) tau, pY^216^GSK3β, GSK3β and β-actin. Representative immunoblotting micrographs are shown on the top panels; the quantitative results are shown on the lower panels. *N* = 2 + 1: Sham: 7, 2K1C: 4; 5 + 1: Sham: 4, 2K1C: 4; 7 + 1: Sham: 3, 2K1C: 4. ^∗^*p* < 0.05, ^∗∗^*p* < 0.01 versus respective Sham group, Bonferroni’s *post hoc* test. Full-length blots are presented in **Supplementary Figure S4**.

One month after the 2K1C treatment, the relative levels of pS412-tau in the hippocampi of 2 + 1, 5 + 1, and 7 + 1 groups were determined. S412-tau was chosen because its phosphorylation was significantly altered after the AAC operation in pigs (**Figure 3A**). It has been demonstrated that tau protein-related pathologies are unevenly distributed in hippocampus layers during the progression of AD and the increasing of the PHF pathology in different layer of hippocampus is correlated to Braak stages and clinical dementia rating scale (Thal et al., 2000). Furthermore, the distribution of the hyperphosphorylated tau protein in different layers of hippocampus was also associated with CERAD diagnosis (Seifan et al., 2015). Therefore, we characterized the effect of 2K1C on the distribution of the different layers in the hippocampus. 2K1C increased the intensities of pS412-tau-positive signals in the stratum radiatum (S.R.) (*F* = 4.5, *p* = 0.046) and stratum lacunosum-moleculare (S.L.M.) (*F* = 8.6, *p* = 0.008) layers of the dorsal hippocampi (**Figure 6A**), and in the stratum oriens (S.O.) (*F* = 7.7, *p* = 0.011) and stratum pyramidale (S.P.) (*F* = 3.3, *p* = 0.048) layers of the ventral hippocampi (**Figure 6B**). Furthermore, the effect of 2K1C on the levels of pS412-tau was affected by age and brain regions. The intensities of pS412-tau signals were comparable in the 2 + 1 and 5 + 1 groups of the dorsal hippocampi; whereas the increased pS412-tau signals were evident in the 5 + 1 groups of the ventral hippocampi. These findings suggest that the ventral hippocampus is more vulnerable to the 2K1C-induced effects than the dorsal hippocampus.

The relative level of pS412-tau in the hippocampi was also determined. Immunoblotting showed that the 2K1C operation significantly increased the level of pS412-tau (*F* = 19.0, *p* < 0.001) but not the total levels of tau (*p* > 0.5) (**Figure 6C**). However, neither the levels of pY^216^GSK3β (*F* = 2.8, *p* = 0.113) nor the total levels of GSK3β (*p* > 0.5) were affected by the treatment (**Figure 6C**).

### 2K1C Impairs Hippocampus-Related Learning and Memory in the 3xTg Mice

**FIGURE 7:**
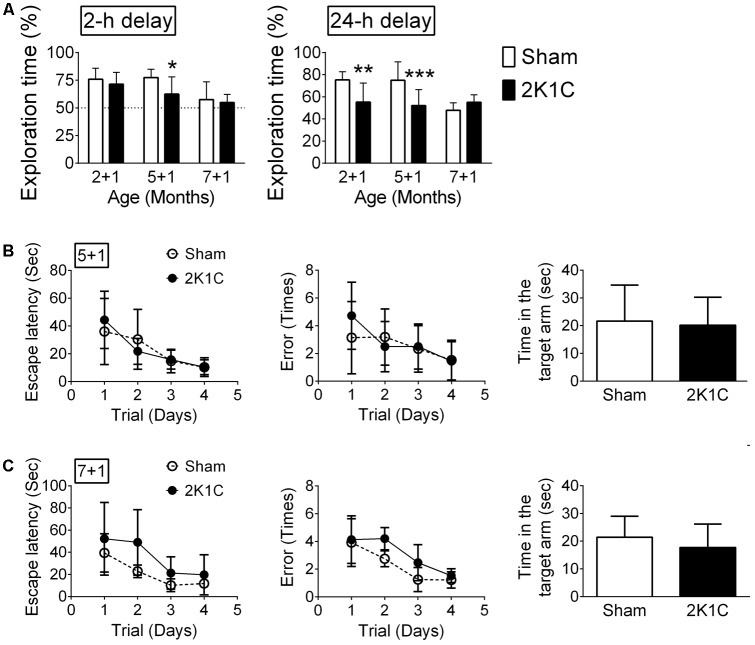
2K1C impairs hippocampus-related learning and memory in the 3xTg mice. 2-, 5-, and 7-month-old mice were given the 2K1C or the Sham operation, and their hippocampal learning and memory were determined at 1 month post-operation (termed 2 + 1, 5 + 1, and 7 + 1 groups). **(A)** Novel object recognition task. The percentages of novel object exploring time (time spent on novel object/time spent on both objects) of short-term (2-h) delay and long-term (24-h) delay. *N* = 2 + 1: Sham: 11, 2K1C: 16; 5 + 1: Sham: 14, 2K1C: 12; 7 + 1: Sham: 8, 2K1C: 8. ^∗^*p* < 0.05, ^∗∗^*p* < 0.01, ^∗∗∗^*p* < 0.001 versus respective Sham group, Bonferroni’s *post hoc* test. **(B,C)** Radial arm water maze. **(B)** 5 + 1 groups. *N* = Sham: 7, 2K1C: 6. **(C)** 7 + 1 groups. *N* = Sham: 6, 2K1C: 7. Escape latency: time to find the hidden platform; Error: total numbers of error made before finding the hidden platform.

The hippocampus-related non-spatial learning and memory ability of mice was evaluated by the object recognition test (Leger et al., 2013). Only mice that did not exhibit a position preference in the task environment were used in this study. In the POM 1 mice (2 + 1, 5 + 1, and 7 + 1 groups), the 2-h delayed short-term memory and 24-h delayed long-term memory were affected by age and 2K1C (**Figure 7A**). Bonferroni’s *post hoc* analyses revealed that the short-term memory of the Sham mice was unaltered until they reached 8-month-old while short-term memory was impaired in 5 + 1 and 7 + 1 2K1C groups (**Figure 7A**). Likewise, the long-term memory of the Sham mice started to decline when they were 8-month-old. However, 2K1C significantly impaired the long-term memory of 3xTg mice as early as 3-month-old. The short-term and long-term memories of the 5 + 3 and 7 + 3 mice in the Sham and 2K1C groups were all impaired (data not shown); our paradigm could not differentiate them.

The hippocampus-related spatial learning and memory ability of mice were evaluated using the radial arm water maze (Shukitt-Hale et al., 2004). In the 5 + 1 groups, neither escape latency (*p* > 0.5) nor the numbers of error made before finding the hidden platform (*p* > 0.5) were affected by 2K1C (**Figure 7B**). In the probe test with the platform removed, the Sham and 2K1C mice spent about the same time in the targeted quadrant (**Figure 7B**). The swim velocity was comparable between two groups (*Mann–Whitney U* = 12, *p* = 0.234). In the 7 + 1 groups, 2K1C marginally increased the escape latency (*F* = 4.7, *p* = 0.038) and the number of errors made (*F* = 4.5, *p* = 0.043). In the probe test, the time spent in the targeted quadrant was comparable between Sham and 2K1C groups (**Figure 7C**). There was also no difference in the swim speed (*p* > 0.5).

### 2K1C Disrupts the Hippocampal BBB Integrity in the 3xTg Mice

**FIGURE 8:**
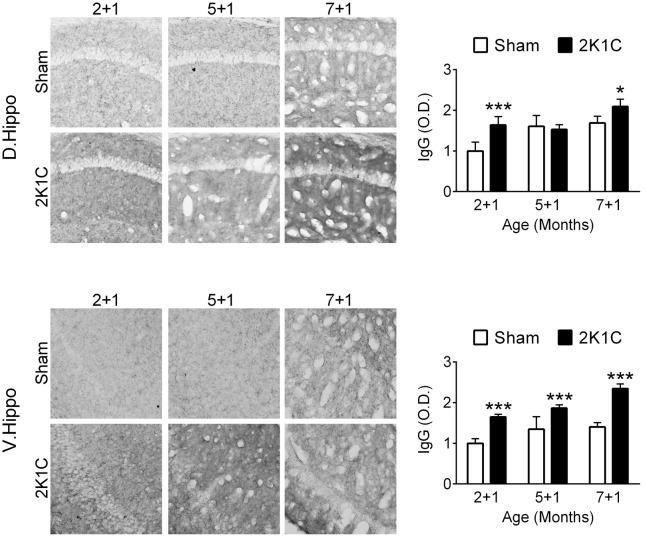
2K1C disrupts the hippocampal BBB integrity in the 3xTg mice. 2-, 5-, and 7-month-old mice were given the 2K1C or the Sham operation, and their hippocampal relative levels of mouse IgG were determined at 1 month post-operation (termed 2 + 1, 5 + 1, and 7 + 1 groups). Representative immunostaining micrographs are shown on the left panels. The quantitative results are shown on the right panels. *N* = 5 for all ages, treatments and regions. ^∗^*p* < 0.05, ^∗∗∗^*p* < 0.001 versus respective Sham group, Bonferroni’s *post hoc* test. D. Hippo, dorsal part of hippocampus; V. Hippo, ventral part of hippocampus.

One month after the 2K1C treatment, the relative levels of mouse IgG in the hippocampi of the three POM 1 (2 + 1, 5 + 1, and 7 + 1) groups were determined as an indication of BBB integrity. Both age (dorsal: *F* = 20.8, *p* < 0.001; ventral: *F* = 32.0, *p* < 0.001) and 2K1C (dorsal: *F* = 19.5, *p* < 0.001; ventral: *F* = 155.8, *p* < 0.001) significantly affected the intensities of IgG-positive signals (**Figure 8**). *Post hoc* analyses revealed that the effects of 2K1C on the intensities of IgG-positive signals were more pronounced in the ventral portion of hippocampi, in which the IgG-positive signals of the 2K1C groups were higher than those of Sham groups in all three ages (**Figure 8**). These findings suggest that 2K1C disrupts the BBB integrity in the hippocampus.

### 2K1C Decreases the Dendritic Complexity of Hippocampal Dentate Gyrus Granule Neurons in the 3xTg Mice

**FIGURE 9:**
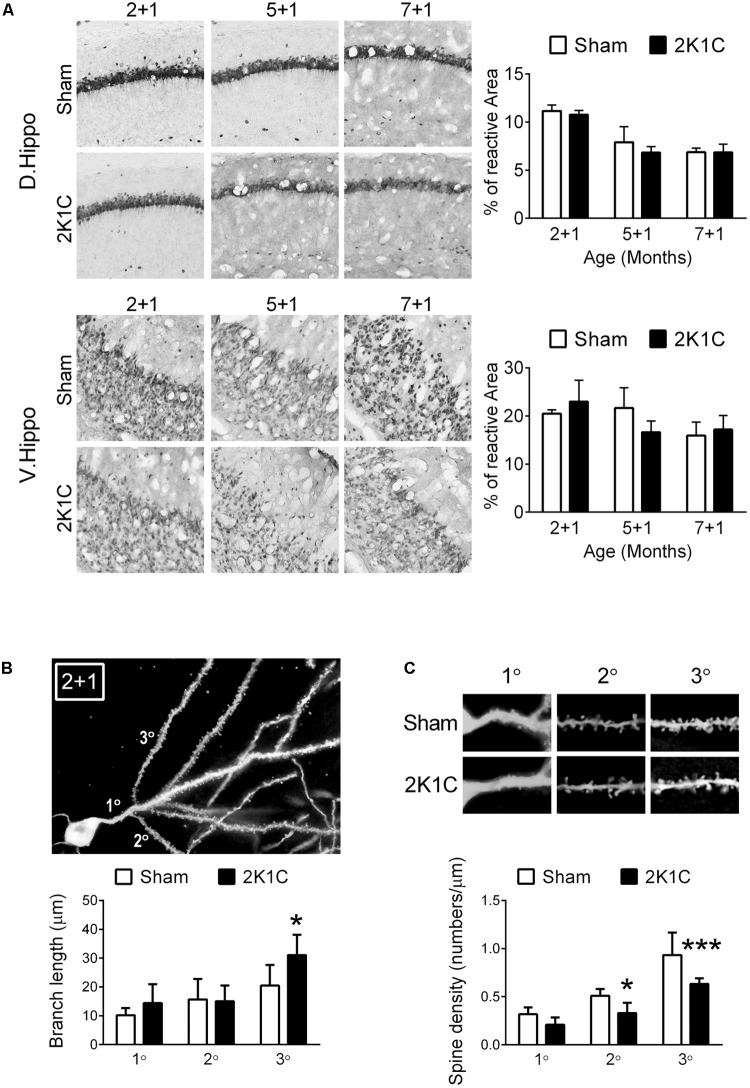
2K1C decreased the dendritic complexity of hippocampal dentate gyrus granule neurons, but not CA1 NeuN-positive area, in the 3xTg mice. **(A)** 2-, 5-, and 7-month-old mice were given the 2K1C or the Sham operation, and their hippocampal relative levels of NeuN were determined at 1 month post-operation (termed 2 + 1, 5 + 1, and 7 + 1 groups). Representative immunostaining micrographs are shown on the left panels. The quantitative results of percentage of NeuN-positive area in dorsal and ventral CA1 region are shown on the right panels. *N* = 5 for each group. D. Hippo, dorsal part of hippocampus; V. Hippo, ventral part of hippocampus. **(B,C)** 2-month-old mice were given the 2K1C or the Sham operation, and the morphologies of hippocampal dentate gyrus granule neurons were determined at 1 month post-operation. **(B)** Dendrite complexity. Representative Golgi staining micrograph is shown on the top panel. Quantitative results of dendritic branch length (distance from soma to branch point) are shown on the lower panel. **(C)** Dendritic spine density. 1°: primary dendrite; 2°: first branch from primary dendrite; 3°: second branch from primary dendrite. *N* = 6 for each group. ^∗^*p* < 0.05, ^∗∗∗^*p* < 0.001 versus respective Sham group, Bonferroni’s *post hoc* test.

The effect of 2K1C on density of pyramidal neurons was determined by percentage of NeuN-positive area in the hippocampal CA1 region (Blanco et al., 2015). Our results showed that age, but not 2K1C, decreased the NeuN-positive areas in the CA1 region of the dorsal and ventral hippocampi (**Figure 9A**).

The Golgi stain was used to investigate the effect of 2K1C on dendritic morphology of the granule neurons in the hippocampal dentate gyrus of the 2 + 1 group. This age group was selected because at this age 2K1C significantly affected the concentrations of Aβ40 and Aβ42 (**Figure 5A**) and the 24-h delayed long-term memory (**Figure 7A**), both are critical indicators for AD pathologies. The branch lengths (distance from soma to branch point) of the 2K1C group were longer than those of the Sham group (*F* = 5.0, *p* = 0.032), particularly in the third order branch (**Figure 9B**). Spine density was positively dependent on branch order (*F* = 59.3, *p* < 0.001), but was negatively affected by 2K1C (*F* = 26.3, *p* < 0.001) (**Figure 9C**). There was no interaction (*F* = 1.9, *p* = 0.162) between branch order and 2K1C on spine density.

## Discussion

Epidemiological evidence suggests there is an association between midlife HTN and late-onset AD. However, whether HTN accelerates the development of late-onset AD or is a distinct disease that becomes more prevalent with age (co-morbidity) remains unclear. By using distinct HTN induction methods in two animal models, we demonstrated that HTN increases AD-related pathologies (hippocampal Aβ concentration, amyloid plaque load and p-tau level), induces shrinkage in dendritic complexity and microglial activation, and moderately impairs learning and memory. Furthermore, by comparing the temporal profiles, HTN accelerates age- and AD gene-related pathological changes and memory impairment. These findings suggest that HTN accelerates the pathogenesis of AD.

The mechanism underlying HTN-induced AD pathogenesis is unclear. It has been suggested that HTN could cause vascular remodeling (e.g., hypertrophic and eutrophic remodeling) to prevent damage from abnormal high blood pressure through increased resistance (Iadecola and Davisson, 2008). However, both types of remodeling lead to a reduction of the vessel lumen. Furthermore, HTN-induced remodeling is known to increase the permeability of the BBB, which then leads to brain inflammation (Wierda et al., 2010). Our results that 2K1C impairs the integrity of BBB and increases microglial activation in the hippocampi support such premise. This process may aggravate atherosclerosis and loss of vessel integrity, which are frequent abnormalities observed in AD brains, and have been correlated with AD pathologies (Beach et al., 2007; Yarchoan et al., 2012). It has been demonstrated that HTN directly reduces the cerebral blood flow (Jennings et al., 2005), which then enhances neuron vulnerability (Zlokovic, 2005).

Aβ concentration in the brain is affected by numerous factors, such as its rates of production, degradation, and clearance. Infusing hypertensive doses of angiotensin II into transgenic APP/PS1 mice has been shown to induce an increase in soluble Aβ levels and accelerate the development of AD-related pathologies (Cifuentes et al., 2015). The levels of β-secretase activity were increased in the angiotensin-II-induced HTN mice, suggesting that HTN may increase the production of Aβ (Faraco et al., 2016). In line with these findings, we showed that AAC-induced HTN increased hippocampal APP levels in the pigs. HTN is known to decrease cerebral blood flow via the downregulation of the endothelial nitric oxide synthase (eNOS)-dependent vessel dilation (Zhou et al., 2004; Cifuentes et al., 2015). Reduction of eNOS activation has been shown to increase the levels of APP in human endothelial cells (Austin et al., 2010). Aβ concentration in the brain is also affected by the equilibrium constant between the brain and its periphery. RAGE and LRP1 are two essential transporters on the blood-brain barrier that mediate the influx and efflux of Aβ peptides, respectively (Shibata et al., 2000; Deane et al., 2003). Our results revealed that the levels of RAGE, but not LRP1, were increased in the hippocampi of AAC-induced HTN pigs. These findings indicate that HTN favors the influx of Aβ from the circulation into the brain. A previous study that adopted the transverse aortic coarctation method to induce HTN in rats also showed an increased level of RAGE in the vascular endothelium cells (Carnevale et al., 2012b). The authors attributed these changes to the HTN-induced inflammation in blood vessels and increased levels of the main ligand of RAGE, advanced glycation end product (AGE) (Carnevale et al., 2012a,b). Although the association among vascular stiffness and AGE formation has been reported (McNulty et al., 2007), it still lacks evidence to support the hemodynamic stress and AGE/RAGE pathway. However, the HTN-induced mechanical stress represents one of the main stimuli for reactive oxygen species generation in vessels (Vecchione et al., 2009), which is known to induce RAGE activation and vascular inflammation (Carnevale et al., 2012b).

Interestingly, the 2K1C-induced elevations of soluble Aβ40 and Aβ42 levels became less pronounced by 8 months old. It is possible that these alterations enhanced the aggregation of soluble Aβ, thus accelerating the formation of amyloid deposition from the typical onset age (i.e., 9-month-old) (Oddo et al., 2003). Therefore, differences in the soluble Aβ levels disappeared while differences in the amyloid loads became evident.

The hippocampus-dependent non-spatial memory seems to be more sensitive to the 2K1C-induced HTN than spatial memory. We did not observe significant changes in the probe trial between the Sham and 2K1C groups. The impairment of hippocampus-dependent non-spatial memory was evident in mice as young as 3-month-old. It is known that the novel object recognition test does not require reinforcers and is purely based on the navigation motivation, while the radial arm water maze test relies on enforcers to drive the spatial navigation (Shukitt-Hale et al., 2004; Leger et al., 2013). Furthermore, the spatial and non-spatial memories appear to be associated with different parts of hippocampal function. The dorsal hippocampus is critical to the formation of spatial memory, while the ventral hippocampus adjusts emotional and motivated related behaviors (Fanselow and Dong, 2010). Lesion in the dorsal hippocampus, but not the ventral hippocampus, induces impairment in the spatial memory of mice during the of radial arm water maze (Pothuizen et al., 2004). In agreement with these findings, levels of p412-tau were already profusely apparent in the ventral hippocampi of 5 + 1 3xTg mice, which was much earlier than those in the dorsal hippocampi that did not show tau-related pathologies until the 7 + 1 group. The temporal-spatial distributions of the tau-related pathologies may explain the temporal variations of the hippocampus-dependent spatial and non-spatial memories. The region-specific phenomenon may be due to the differential density of cerebral vasculatures. The blood vessel density in the ventral hippocampus is higher than that of the dorsal hippocampus, which may make the ventral hippocampus more vulnerable to the HTN-induced effects (Grivas et al., 2003).

The effect of 2K1C on the hippocampus-dependent non-spatial memory were evident at 2 + 1 and 5 + 1 groups, but not the 7 + 1 group. These results may be due to different parameters used in the object recognition test (e.g., size of exploration chamber, color and size of objects, and time of learning phase). For example, the duration of learning object used in this study were 5 min, which are better for testing memory decline from intact states (e.g., 2 + 1 and 5 + 1 groups), not an already impaired state (e.g., 7 + 1 group). When employing such design (5-min learning session) to middle-aged 3xTg mice (i.e., 7 + 1, 5 + 3, and 7 + 3 group), their short-term and long-term memory performance indices were around 50%, indicating that they failed to remember the location of the novel object. Therefore, the 5-min learning session design could not further differentiate the detrimental effect of 2K1C. If we increase the duration of learning object from 5 to 10 min, which will allow the middle-aged 3xTg mice to memorize the location of objects. However, the 10-min design will have a ceiling effect for young mice because they had learned how to do well in the task in 5-min, hence could not resolve the differences between Sham and 2K1C groups of Young 3xTg mice.

The effect of HTN on tau pathology was much more pronounced and consistent than amyloid pathology in both models. In the HTN pig model, we found that AAC increased the hippocampal levels of the active form of GSK3β (pY^216^GSK3β), and decreased the levels of the inactive form of GSK3β (pS^9^GSK3β) without changing the total levels of GSK3β. Activation of GSK3β, also known as tau protein kinase I, has a close relationship with tau phosphorylation (Ishiguro et al., 1993). The changes in the GSK3β levels correspond to the AAC-induced tau phosphorylation. In the mouse model, a strong effect of HTN on tau phosphorylation was also evident, especially in the ventral hippocampus. However, the upregulation of tau phosphorylation was independent of the activity of pY^216^GSK3β and pS^9^GSK3β in the HTN mouse model. Another potential candidate of HTN-associated tau protein kinase is casein kinase 2, which is also capable of phosphorylating the tau protein at ser412 (Hanger et al., 2007). Furthermore, the levels of casein kinase 2 have been shown to increase in the hypothalamus of spontaneously hypertensive rats (Ye et al., 2012). It is possible that HTN upregulates casein kinase 2 in the brain, which then phosphorylates tau.

## Conclusion

In two different HTN induction animal models, we showed that HTN accelerates AD-related pathologies, induces microglial activation and shrinkage in dendritic complexity in the hippocampus, and impairs hippocampus-dependent learning and memory in relatively young animals. The findings suggest that HTN accelerates the onset of AD. Controlling blood pressure especially at midlife represents an important means to delay the onset of AD.

## Author Contributions

Y-HS, M-JJ, and Y-MK were responsible for study design and the idea. Y-HS, S-HH, C-WL, and P-YL carried out the pig study, including the AAC operation, caring, brain preparation, histology staining, and microscopic analysis. Y-HS, S-YW, S-HH, MY, and T-TY carried out the 3xTg mouse study, including 2K1C operation, caring, memory test, brain preparation, histology staining, and microscopic analysis. Y-HS performed the graphic designs and data analysis. T-TY and Y-MK were responsible for budget acquisition, manuscript writing, and final approval. Y-HS, T-TY, and MY helped with manuscript writing.

## Conflict of Interest Statement

The authors declare that the research was conducted in the absence of any commercial or financial relationships that could be construed as a potential conflict of interest.

## Footnotes

1 http://minipigs.angrin.tlri.gov.tw

2 https://www.jax.org/strain/004807

3 http://rsb.info.nih.gov/ij/

## Supplementary Material

FIGURE S1 Abdominal aortic constriction (AAC) increased levels of pT212-tau and pS262-tau in the hippocampal sub-regions of pigs. 7-month-old pigs were given the AAC or the Sham operation, and their expression of pT212-tau and pS262-tau in the hippocampi of POM 3 pigs were analyzed. Representative immunostaining micrographs are shown on the left panels; the quantitative results of relative optical density (O.D.) are shown on the right panel. *N* = Sham: 3, AAC: 3.

FIGURE S2 Full-length blots of APP, RAGE, and LRP-1 in the POM 3 pigs.

FIGURE S3 Full-length blots of pY^216^GSK3β, pS^9^GSK3β, GSK3β, and β-actin in the hippocampi of pigs.

FIGURE S4 Full-length blots of p412-tau, total (Tau-1) tau, pY^216^GSK3β, GSK3β, and β-actin in the hippocampi of 3xTg mice.
