# Supplementary figures and images for: Hypertension Accelerates Alzheimer’s Disease-Related Pathologies in Pigs and 3xTg Mice
Source: Front Aging Neurosci. 2018 Mar 20;10:73. doi: 10.3389/fnagi.2018.00073 (PMC5869211; doi:10.3389/fnagi.2018.00073)

p212-tau

Sham

CA1

CA2

CA3

AAC

□ Sham  
■ AAC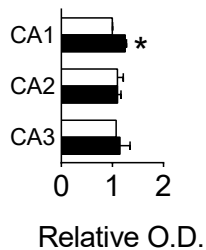

p262-tau

Sham

CA1

CA2

CA3

AAC

□ Sham  
■ AAC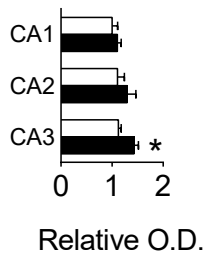

Supplement: FIGURE S1 — Abdominal aortic constriction (AAC) increased levels of pT212-tau and pS262-tau in the hippocampal sub-regions of pigs. 7-month-old pigs were given the AAC or the Sham operation, and their expression of pT212-tau and pS262-tau in the hippocampi of POM 3 pigs were analyzed. Representative immunostaining micrographs are shown on the left panels; the quantitative results of relative optical density (O.D.) are shown on the right panel. N = Sham: 3, AAC: 3. [file Image_1.PDF]

## APP

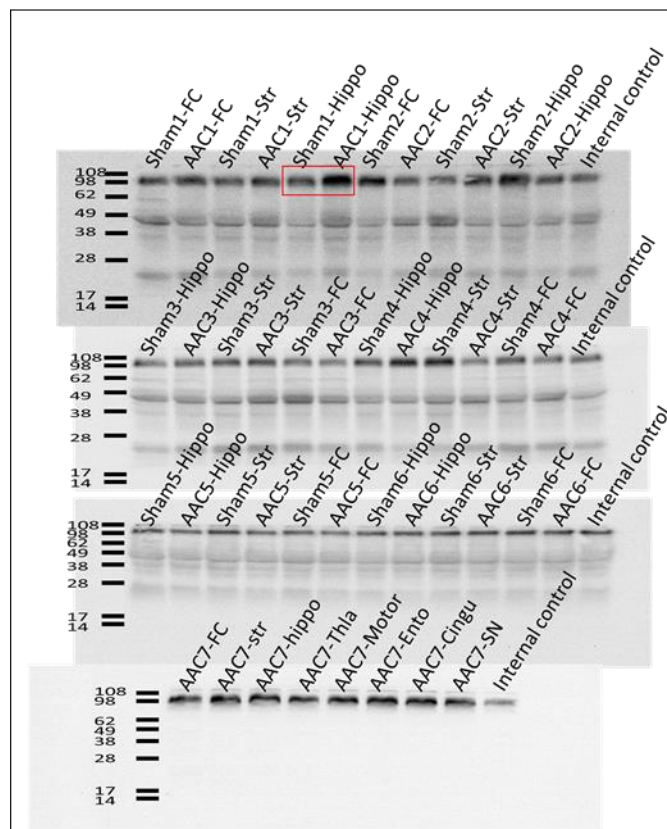

## RAGE

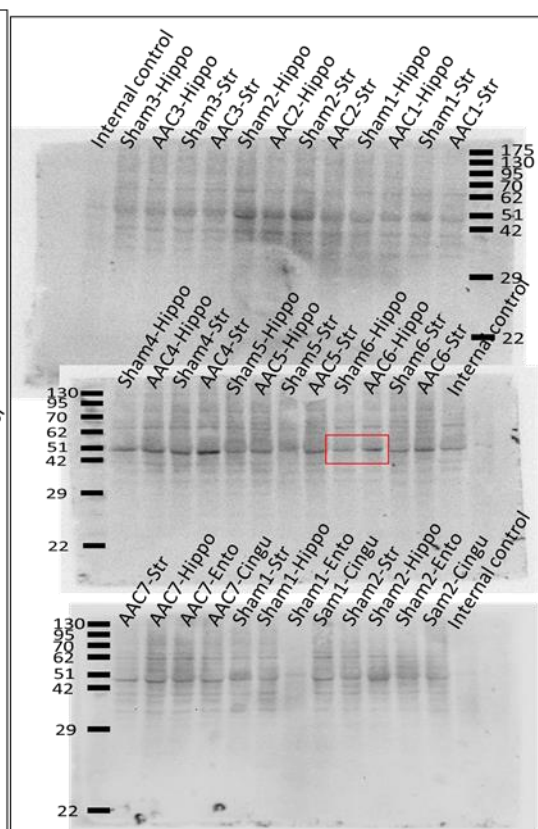

## LRP-1

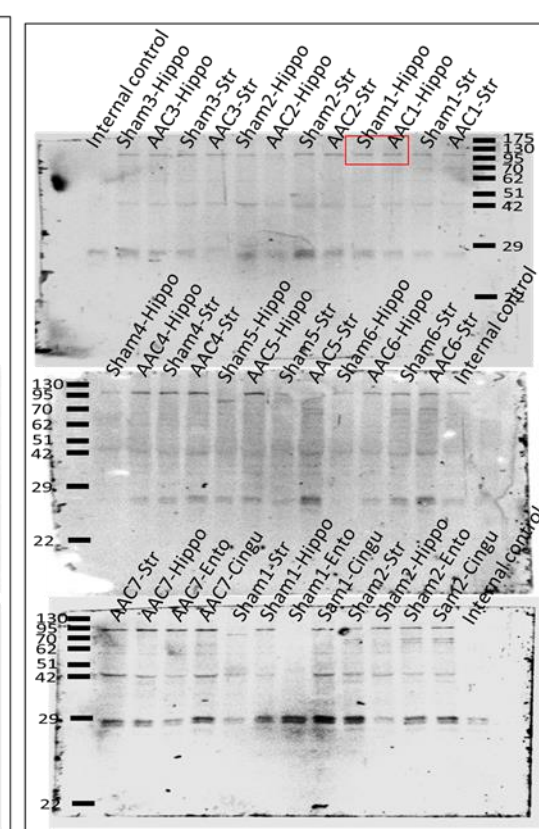

## Actin

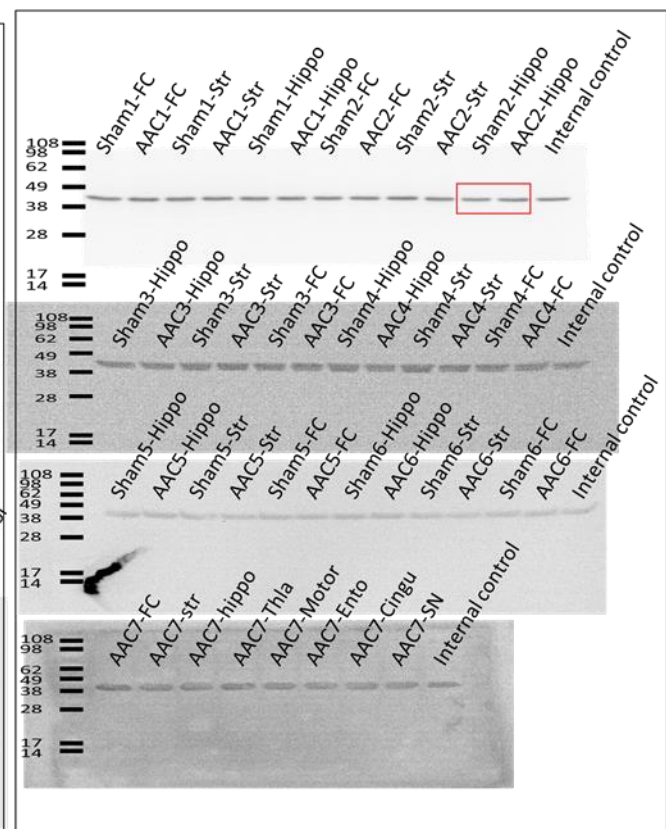

Supplement: FIGURE S2 — Full-length blots of APP, RAGE, and LRP-1 in the POM 3 pigs. [file Image_2.PDF]

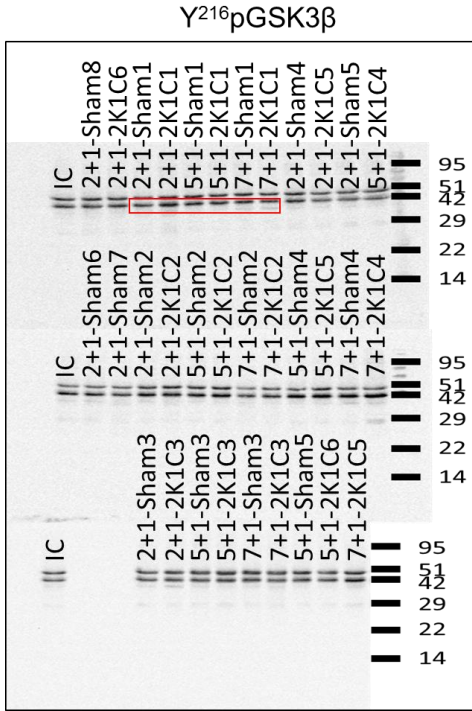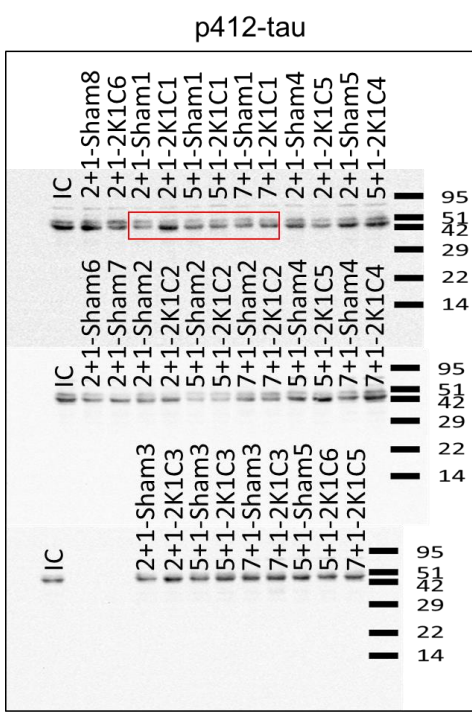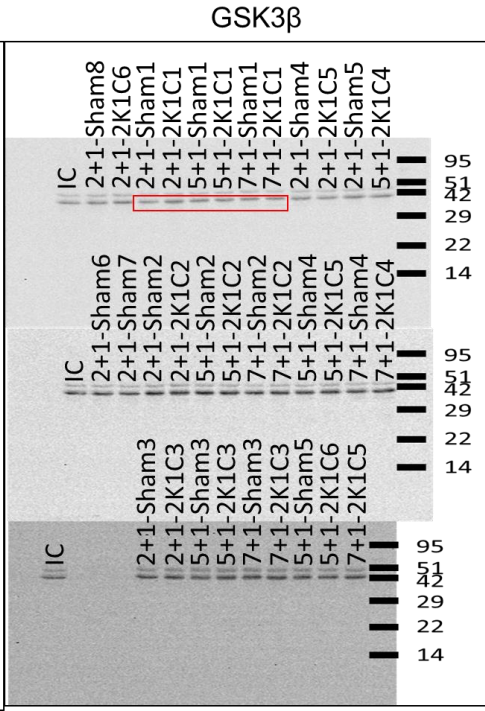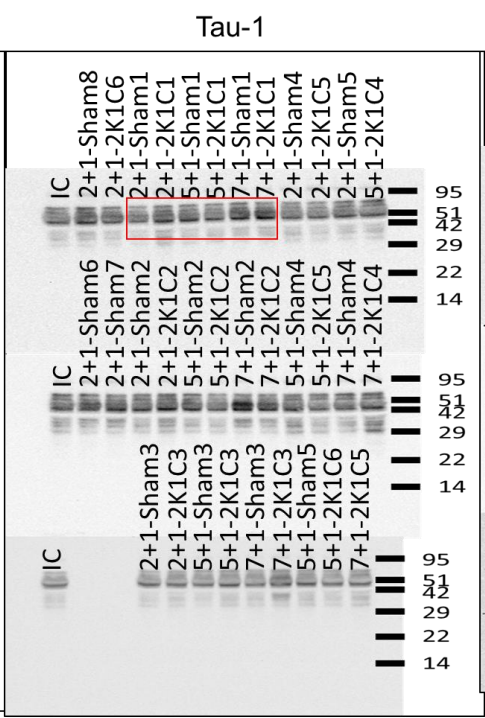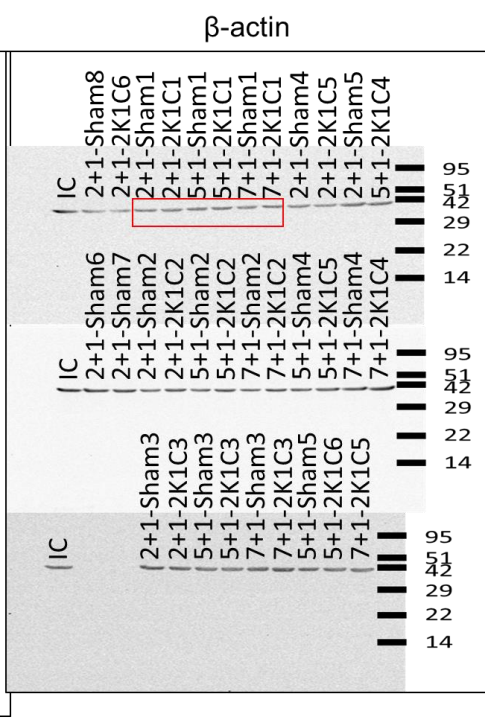

Supplement: FIGURE S4 — Full-length blots of p412-tau, total (Tau-1) tau, pY216GSK3β, GSK3β, and β-actin in the hippocampi of 3xTg mice. [file Image_4.PDF]
