# Supplementary material for: Hypertension Accelerates Alzheimer’s Disease-Related Pathologies in Pigs and 3xTg Mice
Source: Front Aging Neurosci. 2018 Mar 20;10:73. doi: 10.3389/fnagi.2018.00073 (PMC5869211; doi:10.3389/fnagi.2018.00073)
Supplement: FIGURE S3 — Full-length blots of pY216GSK3β, pS9GSK3β, GSK3β, and β-actin in the hippocampi of pigs. [file Image_3.PDF]

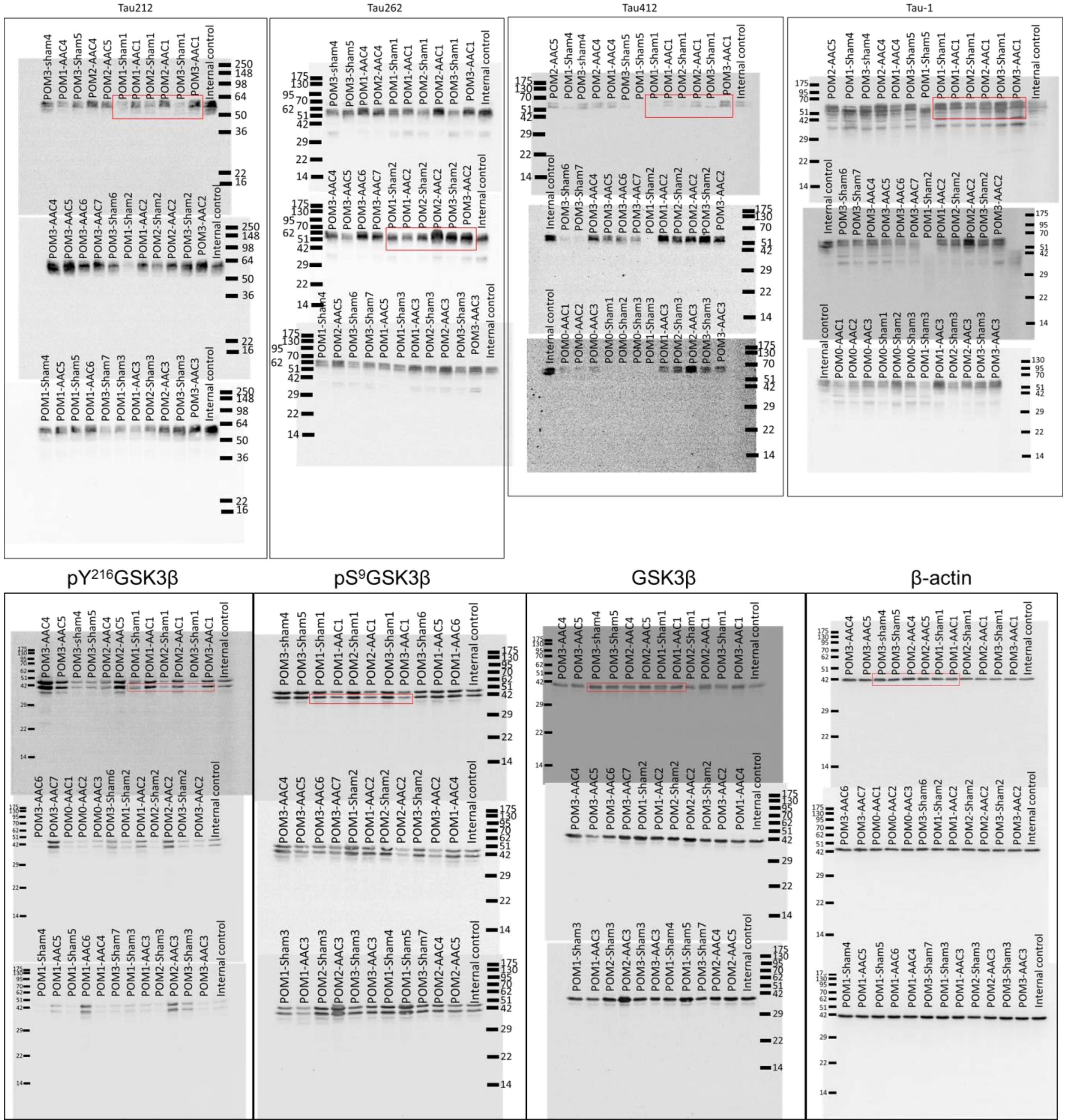
Tau212
Tau262
Tau412
Tau-1
pY216GSK3β
pS9GSK3β
GSK3β
β-actin
